# Impaired T cell receptor signaling in HTLV-1-infected CD4+ cells from HAM/TSP patients

**DOI:** 10.1186/1742-4690-11-S1-P65

**Published:** 2014-01-07

**Authors:** Ryuji Kubota, Hiroshi Takashima, Shuji Izumo

**Affiliations:** 1 Center for Chronic Viral Diseases, Kagoshima University, Kagoshima, Japan; 2 Department of Neurology and Geriatrics, Kagoshima University, Kagoshima, Japan

## 

HAM/TSP patients show increased HTLV-1 proviral load in peripheral blood mononuclear cells (PBMCs), however, little is known about the character of HTLV-1-infected cells. It has been considered that the immune system of HTLV-1-infected individuals are impaired, however, the details are unknown. Here, we investigated HTLV-1-infected cells from HAM/TSP patients for their surface markers and immune function. PBMCs were obtained from the patients and cultured for several hours. HTLV-1-infected cells were identified by detection of intracellular HTLV-1 Tax protein. The Tax-positive, HTLV-1-infected cells showed a phenotype of CD2+CD4+CD5+CD26- CD45RO+CD45RA- CCR4+CCR7- and a reduced expression of T cell receptor (TCR) and CD3 antigen. Next, PBMCs were stimulated with CD3 antibody and TCR signaling was detected by phospho-specific antibodies for Lck and ZAP70, which are early signaling molecules of TCR. The degree of phosphorylation in whole CD4+ cells from HAM/TSP patients were lower than that from normal controls. In addition, Tax-positive CD4+ cells showed reduced phosphorylation of these molecules compared to Tax-negative CD4+ cells in HAM/TSP patients. Finally, cytomegalovirus (CMV)-specific, HTLV-1-infected CD4+ cells showed a decreased production of interferon-gamma by stimulation of CMV antigens compared to CMV-specific, non-infected cells in the patients. These results indicate that HTLV-1-infected cells reduced expression of TCR/CD3 complex in HAM/TSP patients, resulting in a reduction in TCR signaling and impaired immune function.

